# Uses of medicinal plants by Haitian immigrants and their descendants in the Province of Camagüey, Cuba

**DOI:** 10.1186/1746-4269-5-16

**Published:** 2009-05-18

**Authors:** Gabriele Volpato, Daimy Godínez, Angela Beyra, Adelaida Barreto

**Affiliations:** 1 CERES Research School, Department of Social Sciences, Wageningen University, Hollandseweg 1, NL-6706 Wageningen, the Netherlands; 2 CIMAC, Centro de Investigaciones de Medio Ambiente de Camagüey, Cuba. Calle Cisneros No. 105 e/Ángel y Pobre, Camagüey, Cuba

## Abstract

**Background:**

Haitian migrants played an important role shaping Cuban culture and traditional ethnobotanical knowledge. An ethnobotanical investigation was conducted to collect information on medicinal plant use by Haitian immigrants and their descendants in the Province of Camagüey, Cuba.

**Methods:**

Information was obtained from semi-structured interviews with Haitian immigrants and their descendants, direct observations, and by reviewing reports of traditional Haitian medicine in the literature.

**Results:**

Informants reported using 123 plant species belonging to 112 genera in 63 families. Haitian immigrants and their descendants mainly decoct or infuse aerial parts and ingest them, but medicinal baths are also relevant. Some 22 herbal mixtures are reported, including formulas for a preparation obtained using the fruit of *Crescentia cujete*. Cultural aspects related to traditional plant posology are addressed, as well as changes and adaptation of Haitian medicinal knowledge with emigration and integration over time.

**Conclusion:**

The rapid disappearance of Haitian migrants' traditional culture due to integration and urbanization suggests that unrecorded ethnomedicinal information may be lost forever. Given this, as well as the poor availability of ethnobotanical data relating to traditional Haitian medicine, there is an urgent need to record this knowledge.

## Introduction

**Figure 1:**
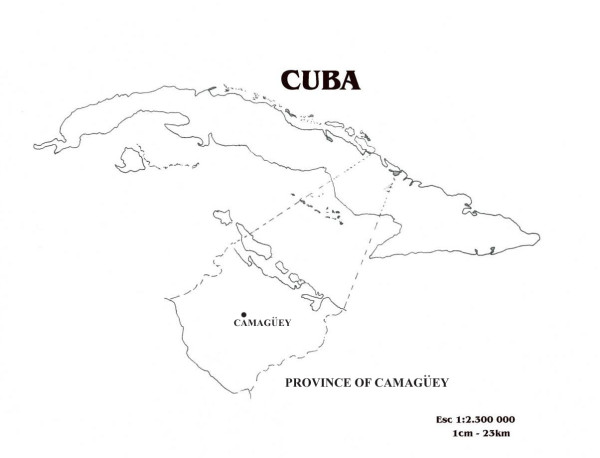
**Map of Cuba with the Province of Camagüey**.

The ethnic and cultural composition of contemporary Caribbean populations are the result of historical population movements through the slave trade and inter-island migration and of the legacy of the different ethnicities involved in the process of national identity formation. Today's Cubans rely for food and medicine on a mixed culture that draws upon wisdom originating mainly from Indian, African, Spanish, and Antillean ethnic groups [1-5]. Among the peoples of African origin who settled in Cuba throughout the centuries, Haitians played an important role shaping Cuban culture and traditional ethnobotanical knowledge. During the period 1900–1930, more than half a million Haitians entered the country legally or illegally [6,7]. Immigration was a key factor in the plans for economic reconstruction after the War of Independence against Spain, and West Indians entered Cuba as cheap labour required to cut sugarcane [8]. Haitians were concentrated in the sugarcane and coffee areas of the former provinces of Oriente and Camagüey (Figure 1). In the latter province, they mainly settled in Haitian communities such as Caidije and Guanamaca, thus permitting the perpetuation of their own culture, including the voodoo religion and the creole language [9-12]. Most Haitians were illiterate, crowded into barracks (*barracones*), paid a miserable salary, and compelled to hand over their savings to reimburse the cost of their passage [7,9]. They relied heavily on homegardens, wild plants, and on traditional ethnobotanical knowledge and practices in order to survive.

Nowadays Haitians are mostly integrated into mainstream Cuban society, although many of them maintain a small-scale farming and livestock production as a base for their livelihoods. Especially over the last decade, Haitians in Cuba have begun to rediscover their roots and revitalize their traditional culture by forming Haitian associations and groups and celebrating festivals and other events. A Haitian carnival takes place every year in Santiago de Cuba, and a Creole radio program is broadcasted nationally [13]. In this context, traditional ethnobotanical practices are sometimes reconstituted as part of Haitian culture [14].

Although in the recent past there has been an increase in ethnobotanical and ethnomedicinal investigations in Cuba [15-19], these have generally not paid attention to the specific ethnic knowledge that immigrants have contributed to traditional Cuban medicine. Consequently, there is little data in the literature about the ethnobotanical knowledge and practices of Haitians in Cuba, with the exception of Volpato et al. [14], while some other information can be found in James et al. [12], Nevet and de la Rosa [9], and Pedro [10]. Moreover, to date only limited data about Haitian traditional medicine has been collected in Haiti, mostly due to the fact that the religious, cultural, and political situation in Haiti has made the study of Haitian ethnomedicine difficult [20]. Exceptions to this are the works of Brutus and Pierre-Noel, León, and Weniger et al. [21-25].

This paper focuses on traditional medicinal plant uses of Haitian immigrants and their descendants in the Province of Camagüey, Cuba. We will present and discuss data about: 1) traditional remedies, their uses, and preparation, 2) traditional practices and beliefs related to these uses, and 3) changes and adaptation of Haitian medicinal knowledge with emigration and integration over time.

## Methods

### Study Area

The Province of Camagüey is located between 20°31'01" and 22°29'00" latitude North and 76°57'00" longitude West from Greenwich. It is located between the Canal Viejo de Bahamas in the North, the Caribbean Ocean in the South, the Province of Las Tunas in the East, and the Province of Ciego de Ávila in the West. Camagüey is the largest province in Cuba, at 15,615 km^2^, corresponding to 14.3% of the nation's territory. The Province is inhabited by some 780,000 people, or seven per cent of the Cuban population. About 75% of the inhabitants live in urban areas, where Camagüey, Florida and Nuevitas are the major cities. About 40% of the total population of the province lives in the city of Camagüey; almost 200,000 people live in rural areas. Due to its mostly flat territory, the Province of Camagüey historically had an economy primarily based on cattle and sugarcane, as well as small-scale farming. Some touristic infrastructures (notably in Camagüey city and Santa Lucía beach) have been developed in the last decade [26].

Although no census of Haitians (residents or descendants) in Cuba has been done to date, we can roughly estimate the number of Haitians and their descendants in the Province of Camagüey at about 50,000 or 6–7% of the population. This figure is based on a comparison with data from another province that also absorbed much Haitian migration to Cuba, the Province of Guantanamo [13]. Creole is the second most spoken language in the Province of Camagüey, after Spanish. Besides Haitians, other ethnic groups in the Province include Jamaicans and Chinese.

### Ethnobotanical investigation

The data presented in this paper are derived from a wider study that was conducted on the ethnobiological knowledge of Haitian people living in the Province of Camagüey. Fieldwork was carried out from December 2002–March 2003 and from February–July 2004. Semi-structured interviews were conducted with 34 Haitians (21 women and 13 men) whose ages ranged from 45 to 102 years (mean age 68), in the following communities: Central Brasil, Jiquí, Aguacate, Esmeralda, Antón, Batey Varela (Antón), San Serapio, Caidije, La Jagua, Macuto 2, Camagüey (neighbourhoods of Puerto Príncipe, Bellavista, Florat, and La Guernica). Most of those interviewed are elderly people living in remote rural areas; they often live alone since, because of their age, their husbands and wives have passed away and their children, if any, have migrated mainly to major Cuban cities (e.g. Camagüey, La Habana). Otherwise, they live in hospices either in Camagüey or in smaller cities and villages.

To locate the respondents, we first focused on the areas in the province where historical and oral records indicate the presence of Haitian communities (e.g. around Central Brasil, Minas in the North of the Province and Central Haiti in the South). Once in the field, we asked for the help of the local government officers responsible for health (doctors or nurses from the local hospital) to determine whether there were any elderly Haitians living in the locality and precisely where. Respondents in the city of Camagüey were located thanks to the local Haitian Association.

Among the Haitians interviewed, 21 migrated to Cuba between 1913–1926, ten are the offspring of Haitian couples who entered Cuba during the same period, and three more left Haiti between 1946–1954. Among first generation migrants, twenty are originally from the cities of Les Cayes (Creole name Okai) and Port Salut (Creole name Posalí), in the South of Haiti, whereas four lived in or near Port-au-Prince. People who migrated in the 1920s generally sailed to eastern Cuba looking for jobs on the sugarcane plantations to improve their living conditions and support their families in Haiti. Those who arrived in the 1940s came either by plane or boat, although they were migrating mostly for the same reasons.

Interviews were conducted in Spanish after first explaining the aims of the study. Throughout the field study, the ethical guidelines adopted by the American Anthropological Association [27] were followed. The plants cited were photographed, collected with the informants during the interviews, and identified by authors (D.G., A.B., A.B.) following León [28], León and Alain [29-31] and Alain [32,33]. Voucher specimens were deposited at the CIMAC herbarium in Camagüey (HACC).

## Results and discussion

### Plants used, preparation and medicinal application

Additional file 1 lists the plant species cited by informants in alphabetical order according to their scientific name, along with their botanical families, vernacular Cuban and Haitian names (as reported by informants during the fieldwork), voucher specimen numbers, parts used, preparation of the remedies, medicinal use, and frequency of mention. Mixtures (components, parts used, preparation and means of use) are given in Table 1, whereas the presence of species in mixtures is reported in Additional file 1. The research led to the identification of 123 different plant species used for medicinal purposes by Haitians and their descendants in the Province of Camagüey. The species belong to 112 genera and 63 families, with a prevalence of *Annona *and *Citrus *(three species each) among the genera, and among the families of Fabaceae (9.8%), Asteraceae (6.5%), Euphorbiaceae and Verbenaceae (4.9%), Lamiaceae and Rutaceae (3.3%).

**Table 1:** Herbal mixtures used by Haitian immigrants and their descendants in the Province of Camagüey

**No**.	**Species (part used^a^) and other products**	**Preparation**	**Way of use**	**Medicinal use**
1	*Cissus verticillata *(ap), *Bidens pilosa *(ap), *Eupatorium odoratum *(sh), *Pluchea carolinensis *(le), *Citrus aurantiifolia *(fr ju), sugar	decoction is filtered	ingestion a a syrup	fever, catarrh

2	*Cissus verticillata *(ap), *Phyllanthus procerus *(ap)	decoction	ingestion	fever, catarrh

3	*Cissus verticillata *(ap), *Terminalia catappa *(le), *Piper aduncum *(le)	decoction	ingestion	fever, catarrh

4	*Protium cubense *(le), *Bidens pilosa *(ap)	decoction	ingestion	catarrh, influenza

5	*Cissus verticillata *(ap), *Petiveria alliacea *(le)	decoction	ingestion	catarrh

6	*Cucurbita maxima *(le), *Persea americana *(le), *Annona squamosa *(le)	decoction	ingestion	*empacho*

7	*Commelina elegans *(le), *Chrysophyllum oliviforme *(le), *Majorana hortensis *(le)	decoction	ingestion	nervous problems

8	*Psidium guajava *(sh), *Gliricidia sepium *(sh), *Citrus aurantiifolia *(sh)	decoction	ingestion	catarrh

9	*Rheedia aristata *(ba), *Cuminum cyminum *(se), fat of *majα *(*Epicrates angulifer *Bibron, Boidae)	plant parts are fried in the fat	ingestion	asthma

10	*Zingiber officinale *(rh), *Citrus aurantiifolia *(fr), *Cymbopogon citratus *(le), *Citrus aurantium *(fr peel), sugar	decoction	ingestion	catarrh

11	*Desmodium *spp. (ap), *Jatropha gossypifolia *(ro), *Erythroxylum havanense *(ro), salt	decoction	ingestion	ovary pain

12	*Crescentia cujete *(in fr), *Cecropia schrebiana *(le), *Cissus verticillata *(ap), *Hibiscus elatus *(fl), sugar	cook, triturated and stirred	ingestion	*miel de güira*: asthma

13	*Crescentia cujete *(in fr), bee's honey, rum, sugar	cook, triturated and stirred	ingestion	*miel de güira*: catarrh

14	*Crescentia cujete *(in fr), *Allium sativum *(bu), *Canella winterana *(ba), *Myristica fragrans *(se), *Pimenta dioica *(se), *Cymbopogon citratus *(ro), *Illicium verum *(fr), rum, sugar	cook, triturated and stirred	ingestion	*miel de güira*: catarrh, stomach pains

15	*Crescentia cujete *(in fr), bee's honey	cook, triturated and stirred	ingestion	*miel de güira*: catarrh, intestinal parasites

16	*Crescentia cujete *(in fr), *Zingiber officinale *(rh), bee's honey, rum	cook, triturated and stirred	ingestion	*miel de güira*: 'cold uterus', female infertility

17	*Bursera simaruba *(sh), *Annona squamosa *(ro)	decoction	ingestion	catarrh

18	*Acalypha alopecurioides *(le), *Isocarpha atriplicifolia *(ap)	decoction or infusion with salt	ingestion	*empacho*, stomach problems

19	*Brachiaria purpurascens *(ro), *Cajanus cajan *(ap)	decoction	ingestion	intestinal parasites

20	*Capraria biflora *(sh), *Parthenium hysterophorus *(ro)	decoction of three shoots and three roots	ingestion	colds, inflammations, ovaries pain

21	*Jatropha gossypifolia *(ro), *Erythroxylum havanense *(ro)	decoction	topical application	oedemas

22	*Justicia pectoralis *(ap), *Lawsonia alba *(le)	decoction	ingestion	headache, nervous problems

^a ^Part(s) used: ap, aerial part; ba, bark; bu: bulb; ep, fruit epicarp; fl, flowers; fr, fruits; ft, flowering tops; la, latex; le, leaves; ls, leaf stalks; re, resin; rh, rhizome; ro, root/tuber; se, seeds; sg, stigma; sh, shoots; st, stems; uf, unripe fruits; wh, young whorls; wo, wood; wp, whole plant.

The most frequently used species are *Chenopodium ambrosioides*, *Cissus verticillata*, *Cocos nucifera*, *Crescentia cujete*, *Cymbopogon citratus*, *Lippia alba*, *Momordica charantia*, *Pimenta dioica*, *Portulaca oleracea*, *Psidium guajava*, and *Stachytarpheta jamaicensis*. The plant parts used include: leaves and aerial parts (53.5% as a whole), young leaves and shoots (9.7%), seeds and fruits (8.4% each), roots and tubers (7.7% as a whole), bark (4%), stems (3%), flowers (2.3%), rhizomes (1.3%), and resins and bulbs (0.6% each).

The decoction of fresh herbal components is by far the preferred means to prepare medicinal remedies, accounting for almost 60% of all preparations, which is similar to what has been found in traditional Cuban medicine [15,17,19]. Given the availability of medicinal plants in the surrounding environment, for some species at least, the use of fresh plants may present the advantage of preserving more active compounds and consequently enhancing their absorption and effectiveness. About 10% each of the remedies are prepared by means of juice extraction and infusion. Generally, decoction is used for hard and ligneous parts, including coriaceous leaves, while infusion is used only for soft leaves and shoots, especially from aromatic plants (e.g. *Bidens pilosa*, *Cymbopogon citratus*, *Majorana hortensis*, *Ocimum *spp.). Juice extraction is mostly used for green parts and is preferred over decoction and infusion for topical applications. Remedies prepared by heating plant parts in fire (four per cent) are mostly used for topical applications (e.g. leaves applied to the forehead to treat headache). The relatively high figure for alcoholic maceration (8.7%) is due to the number of plants that are reported to be soaked in rum and used in the preparation of a medicinal and ritual Haitian drink called *tifey *[14]. Almost five per cent of the remedies are used without processing, which is especially the case for fruits eaten as medicinal foods (e.g. *Psidium guajava *as an anthelmintic).

Ingestion is the preferred means to administer the remedies and accounts for 62% of all applications. Topical application as a pomade or plaster is used in 10% of the remedies, while frictioning, preferred with preparations for rheumatisms and arthritis, accounts for two per cent. Baths are the second more important category of means of application at almost 16% of the total. They are used to treat rashes in children caused by measles and smallpox (e.g. with *Momordica charantia*, *Hamelia patens*), as well as to treat skin infections such as carbuncles, to alleviate itching, and to fortify children who have 'fragile health'. Children's baths prepared with anthelmintic plants (e.g. *Chenopodium ambrosioides*, *Momordica charantia*) are used to treat intestinal parasites. Often, a decoction of leaves and aerial parts is prepared, sometimes in combinations of different species, and left to cool, or otherwise these vegetal parts are smashed and directly added to the bath water. Baths are also prepared to rid people of the 'bad' and the 'evil eye', a practice known in Afro-Cuban religions as *despojo *[34,35], mainly using species such as *Vitex trifolia, Trichilia glabra, Alpinia speciosa, Allophyllus cominia*. Often this practice is associated with a ritual acknowledgement of the plant and its power, by leaving a coin in the place where leaves have been collected, or by adding a coin to the bath and later leaving it at road crossing. In the case of a child with persistent 'evil eye' (for example when the child cries excessively), after the bath the child's clothes are burnt, and a collar is made with seeds of *Canavalia ensiformis *and placed on the child, as reported also in Haiti [36]. The practice of using herbal baths both as physical and spiritual medicine is similar to other ethnic groups [37,38]; as well, baths are very important in general in traditional health systems based on Afro-American religions [39], and their use among Haitians can be regarded at the same time as magical, spiritual, and medicinal.

In reference to therapeutic use, almost half of the remedies are intended to treat gastro-intestinal afflictions (stomach pains, and as digestive and carminative; about 20%) and afflictions of the respiratory system (catarrh, asthma, colds, cough; about 18%). Other therapeutic uses treat afflictions of the reproductive apparatus (menstrual disorders, ovary pain, vaginal infections, as an aphrodisiac; about 9%), skin afflictions (wounds, burns, rashes; about 9%), helminth worm infections (about 7%), and renal afflictions (diuretic, depurative; about 7%).

A few other remedies of non-vegetal origin were also reported. Goat feces are dried, powdered, mixed with olive oil and applied topically for burns, while packages made of urine and cotton are applied to the back of the heads of children with fever. Also, cricket's (genus *Acheta *and *Neoconocaephalus*) legs are boiled in water and the decoction is then drunk by children and older people who have urination problems. This use of cricket's legs has been also reported by Hernández and Volpato [19] in their article about the medicinal mixtures of Eastern Cuba, as well as by Seoane [16] in his treatise on Cuban medical folklore.

### Herbal mixtures

Besides single medicinal plants, informants also reported 22 herbal mixtures that are mostly prepared as a concoction of plants or plant parts and ingested. These mixtures can be more or less complex, ranging from a concoction of two plants to complex preparations with different species. More than 50% of the mixtures are used to treat afflictions of the respiratory system. Five formulas have been reported as *miel de güira *(*siwò kalbaz *in Creole), whose main ingredient is the fruit of *Crescentia cujete*. In its basic preparation, the inner mass is cooked, triturated, and then stirred, sometimes being left one night outside of the house before stirring. The resulting juice is then mixed with sugar and/or bee's honey and sometimes a small amount of rum, and drunk/eaten for problems of the respiratory system (asthma, catarrh), of the digestive system (stomach pains, intestinal parasites), and of the female reproductive apparatus (infertility) [19]. Different plant species are added to the basic preparation according to the specific medicinal purpose for which it is prepared: for example, *Cissus *spp. and *Bidens pilosa *are added to treat congestions of the respiratory system, whereas 'hot' plants (e.g. *Canella winterana*, *Pimenta dioica*) are added to preparations with stomachic purposes. *Miel de güira *is considered as a panacea, and its use is apparently widespread among Cuban and Cuban-Haitian populations as a preventive and a remedy, when it is taken in small spoons in doses of from one to five spoons per day [16].

### Traditional posology of the remedies

The complexity of practices related to traditional posology is rarely investigated in ethnobotanical and ethnopharmacological studies. Among Haitians, these practices are often related to cosmological/ritual numbers, and plant quantities used in the preparation of the remedies and the timing of administration follow these numbers (mainly *three *and *seven*; see also Weniger et al. [25]). For example, *three *shoots of *Mangifera indica *are boiled and the remedy is drunk in *three *different cups to treat *empacho*, a digestive problem; *three *leaves of *Cissampelos pareira *are split into half and *three *halves are boiled in the case of fever; an infusion made from *three *whorls or tops of *Stachytarpheta jamaicensis *is prepared and given to children in the morning on an empty stomach as an anthelmintic; the decoction of *three *leaves of *Momordica charantia *must be drunk for *three *days, and the seeds of the same plant are ingested one on the first day, two on the second, and three on the third, and so on for *seven *days. Rituality based on 'sacred' numbers represents, in these cases, a simple way of memorizing the proper dose to be used, as well as a contribution to the efficacy of the remedy by calling upon supernatural forces and entities related to those numbers.

Haitian's knowledge about plants seems to comprehend and deal with toxic allelochemicals through specific posological practices. For example, a small spoonful of the hairs of the fruits of *Mucuna pruriens *is mixed with *Psidium guayaba *jam and ingested before breakfast for three days; the massive diarrhea that follows is supposed to eliminate all worms from the gut and the stomach, as reported also by Seoane [16]. The hairs of the fruit of this plant contain formic acid and mucunain, which are so toxic that they were used as homicidal poisons in Africa [40,41]. Also, in the anthelmintic use of *Chenopodium ambrosioides*, we can distinguish a posology for acute episodes (*three *buds every day before breakfast for *three *or *seven *days), and a posology for chronic infection (e.g. only with the new moon [42]), where the remedy is ingested periodically throughout the year. The continuous ingestion of low doses of the allelochemicals in these species may be an effective means to prevent massive parasite infestations, especially in children [43].

### Changes in and adaptation of medicinal plant use with emigration and cultural integration

Ethnobotanical knowledge is dynamic for any given culture and it changes as it is transferred and appropriated by people who are adapting to new environments [44,45]. Ethnomedicinal knowledge of Haitian immigrants in Cuba presents no exception [14]. Migrants confront a different sociocultural context and new environments where specific plants may no longer be available and traditional practices may come under pressure and therefore may be progressively adapted or abandoned [46]. In these contexts, the main forces that drive change in the cultural domain of traditional medicinal knowledge are: (1) the adaptation of the original knowledge to the new (host) environment (through substitution of no longer accessible traditional remedies with locally available ones, and the incorporation of remedies from the host culture into migrants' own pharmacopoeia); and (2) the development of strategies to obtain the original remedies (through cultivation, gathering, or marketing of the original remedies, and the development of social networks that link migrants to relatives and friends in the place of origin) [47,48].

In this article we have presented the medicinal plants' knowledge of Haitians in Cuba as it is today, approximately 80 years after migration. This lapse of time is long enough to permit insights to be drawn regarding the process of transformation and adaptation of ethnomedicinal knowledge after migration and in the ways in which the progressive integration of migrants in the host culture modifies this knowledge. To gain further insights, we qualitatively compared our results with those reported in other Cuban ethnobotanical studies [18,19,42,49] and especially with the work of Beyra et al. [15] who interviewed 29 Cuban informants across the Province of Camagüey and reported 111 species used for medicinal purposes. More than half of the plant species reported in that study are also reported in the current study of Haitian immigrants and their descendants. Of these, about three quarters were reported with the same medicinal uses, and the remaining quarter with different uses. Among those plants with shared uses are species that are widely used in Cuban pharmacopoeia such as *Bidens pilosa*, *Boldoa purpuracens*, *Phyla scaberrima*, *Pluchea carolinensis*, and *Rheedia aristata*, whose medicinal uses may have partly been adopted by migrants, as well as medicinal plants that are common to the Caribbean pharmacopoeia whose use Haitians and Cubans shared prior to migration: examples include the use of *Cecropia schrebiana *as an anticatarrhal; of *Carica papaya*, *Chenopodium ambrosioides *and *Psidium guajava *to treat intestinal parasites; of *Lepidium virginicum *as a carminative and diuretic; and of *Zingiber officinale *to treat colds, catarrh, and rheumatic pains. Some plant uses have a common origin in the ethnobotanical practices of Caribbean people of African cultural heritage, the so-called Afro-Caribbean pharmacopoeia: examples include the use of the aerial parts of *Lippia alba *and *Cymbopogon citratus*, as well as the use of roots and ligneous parts of *Allophylus cominia*, *Caesalpinia bahamensis*, *Erythroxylum havanense*, and *Chiococca alba*. Afro-Caribbean pharmacopoeia is that body of knowledge and practices around medicinal plants which finds its origins in the cultures of African slaves brought to the Caribbean [50]. So, *Lippia alba *and *Cymbopogon citratus *often appear in the corpus of ethnobotanical knowledge of African origin in Cuba [14,51], and *Erythroxylum havanense *and *Chiococca alba *are among the main ingredients of multi-herbal preparations used as a medicinal remedy in Eastern Cuba as well as a spiritual remedy in Afro-Cuban religions [19,34].

Other medicinal uses reported in this study and also commonly found in the Cuban pharmacopoeia include the use of the aerial parts of *Cissus verticillata *for respiratory problems, of the young fruit of *Cocos nucifera *and the leaves of *Portulaca oleracea *for intestinal parasites, of the bark and the leaves of *Mangifera indica *for gastrointestinal and respiratory problems respectively. Among the shared ethnobotanical practices is also the preparation of *miel de güira *with the pulp of the fruit of *Crescentia cujete*. Remedies shared between Haitian immigrants and their descendants and the Cuban population are mainly the result of the presence of shared ethnobotanical knowledge before migration took place, but as well reflect adoption by Haitian immigrants of plants and/or uses from the dominant Cuban pharmacopoeia and, to a lesser extent, *vice versa*. In contrast, the use of the same species with different medicinal purposes may be the result of migrant's adoption of some species through experimentation with plants found in the new environment (e.g. the use of *Dichrostachys cinerea *as antidiarrhoeic) or incomplete imitation of local practices. Data also suggest that culturally relevant plants (those cited by more informants and with a greater number of uses) are often used in different qualitative ways by migrants and hosts. *Momordica charantia*, *Solanum americanum *and *Stachytarpheta jamaicensis *are among those species most cited by Haitians in this study. Although they are also reported in Beyra et al. [15] and in other studies about traditional Cuban medicine [18,42], their use among Cubans is not as widespread or as differentiated as among Haitian descendants. Conversely, *Justicia pectoralis*, reported by Haitians only as a component of one mixture, is widely used and reported by Cubans for its sedative effects [15,19].

Almost half of the plants reported in this study are not reported in Beyra et al. [15]. Among these, there are plants that are important medicinals for Haitians, such as *Artemisia absinthium*, *Phyllanthus procerus*, and *Priva lappulacea*, as well as culturally relevant Haitian food plants that are also used in the realm of traditional medicine, such as *Abelmoschus esculentus*, *Cajanus cajan*, *Corchorus siliquosus*, and *Xanthosoma sagittifolium*, and some species used for ritual and religious baths such as *Allophylus cominia*, *Alpinia speciosa*, and *Vitex trifolia*. Although medicinal uses of these plants are not absent from the Cuban pharmacopoeia, they may in some cases be restricted to Haitian descendants and to Cubans who have been influenced by the migrants' culture.

**Figure 2:**
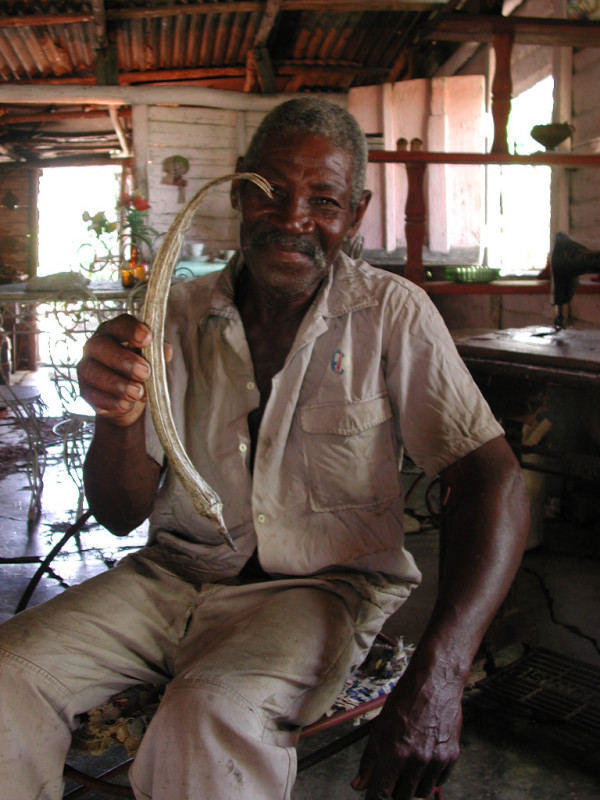
**Haitian with a dried fruit of *Abelmoschus esculentus *from his homegarden (G. Volpato)**.

For most Haitian migrants, given their poverty, there was no possibility to make trips back to Haiti to procure remedies that were not available in the new environment. Nevertheless, some culturally relevant products such as dried or fresh specimens of *Artemisia absinthium *and fruits and seeds of *Abelmoschus esculentus *were brought to Cuba upon migration (Figure 2). Once they found themselves in Cuba, the main strategies that Haitian migrants used to maintain their ethnomedicinal practices depended principally on the floristic similarity between Haiti and Cuba (i.e. most plants used in Haiti were also available in Cuba), and to the cultivation of medicinal plants in the new environment. The incorporation of local remedies into their own pharmacopoeia occurred as a consequence of factors such as cultural contacts and exchanges between Haitians and Cubans and of personal experimentation or imitation of local practices by migrants. Conversely, and to a lesser extent, Haitians contributed to what is today considered as traditional Cuban medicine by introducing into the dominant Cuban community certain specific ethnobotanical practices and uses of plants, as described also in Volpato et al. [14].

## Conclusion

The present investigation shows that Haitian migrants and their descendants living in the Province of Camagüey (Cuba) have medicinal uses for 123 plant species belonging to 112 genera in 63 families. The most frequently used species are *Chenopodium ambrosioides*, *Cissus verticillata*, *Cocos nucifera*, *Crescentia cujete*, *Cymbopogon citratus*, *Lippia alba*, *Momordica charantia*, *Pimenta dioica*, *Portulaca oleracea*, *Psidium guajava*, and *Stachytarpheta jamaicensis*. Decoction of fresh herbal components (mainly leaves and other aerial parts) is the preferred means to prepare medicinal remedies. Herbal baths are important in Haitian culture in both spiritual and medicinal practices, and represent the second most important category of administration, after ingestion. Besides single medicinal plants, 22 herbal mixtures, mostly prepared as a concoction of plants or plant parts, are reported. Among these, a mixture prepared with the fruit of *Crescentia cujete *as a main ingredient is highly regarded by Haitians and is considered as a panacea.

Haitian ethnobotanical practices related to traditional posology often follow cosmological/ritual numbers, both for plant quantities and timing of administration. At the same time, posology is embedded in specific rituals that are performed during the preparation of the remedies, which on the one hand serve to memorize the proper dose, especially when dealing with toxic allelochemicals, and on the other hand contribute to the efficacy of the remedy by invoking supernatural forces and entities related to those rituals and numbers. Traditional and ritual plant posology should be investigated in more depth in ethnobotanical and ethnopharmacological studies in order to understand their relation with medicinal plant efficacy and toxicity.

Traditional Haitian medicine retained an important role in healthcare and cultural practices soon after immigration, when Haitian livelihoods were based on work in the sugarcane fields, on the surrounding environment, and on their knowledge about that environment. During the decades after emigration, the original Haitian ethnomedicinal knowledge progressively changed and adapted to the new environment, maintaining cultivation and use of important medicinal plants, incorporating plants and uses from the host Cuban culture, and diffusing specific plant uses to Cubans in contact with Haitian communities. The study of Haitian immigrants' traditional medicine in this context not only represents an interesting case about medicinal plant use, but also records knowledge that is rapidly disappearing with the death of older Haitian migrants.

## Competing interests

The authors declare that they have no competing interests.

## Authors' contributions

GV and DG conceived and designed the research. GV, DG, AB, and AB carried out interviews and collected data in the field. DG, AB, and AB performed botanical analysis and species identification. GV drafted the manuscript. All authors read and approved the final manuscript.

## Supplementary Material

Additional file 1 **Medicinal plants used by Haitian immigrants and their descendants in the Province of Camagüey, Cuba**. Inventory of medicinal plants used by Haitian immigrants and their descendants in the Province of Camagüey, Cuba. Scientific name, botanical family, vernacular Cuban and Haitian name(s), voucher specimen number, part(s) used, preparation, use(s), and frequency of mention are reported for 123 plant species used for medicinal purposes. Click here for file
