# Supplementary material for: Uses of medicinal plants by Haitian immigrants and their descendants in the Province of Camagüey, Cuba
Source: J Ethnobiol Ethnomed. 2009 May 18;5:16. doi: 10.1186/1746-4269-5-16 (PMC2690575; doi:10.1186/1746-4269-5-16)
Supplement: Additional file 1 — Medicinal plants used by Haitian immigrants and their descendants in the Province of Camagüey, Cuba. Inventory of medicinal plants used by Haitian immigrants and their descendants in the Province of Camagüey, Cuba. Scientific name, botanical family, vernacular Cuban and Haitian name(s), voucher specimen number, part(s) used, preparation, use(s), and frequency of mention are reported for 123 plant species used for medicinal purposes. [file 1746-4269-5-16-S1.pdf]

Additional file 1. Medicinal plants used by Haitian immigrants and their descendants in the Province of Camagüey, Cuba							
**Botanical taxon (voucher specimen)**	**Botanical family**	**Cuban phytonym[a]**	**Haitian (Creole) phytonym[a]**	**Parts used[b]**	**Preparation[c] and way of use[d]**	**Medicinal use**	**Quotation frequency[e]**
Abelmoschus esculentus (L.) Moench	Malvaceae	quimbombó	kalalou	se	7 or 21 seeds mac in rum, ing	aphrodisiac	●
				fr	mac in rum, ing	refreshing and diuretic	●
Acalypha alopecurioides Jacq. (8455)	Euphorbiaceae		degonflé	le	dec or inf with salt, ing	*empacho*, stomach pains and digestive problems	●●
					mix 18	mix 18	
Allium sativum L.	Liliaceae	ajo	lay	bu	tr, ing	intestinal parasites	●●
					mix 14	mix 14	
Allophyllus cominia (L.) Sw. (8603)	Sapindaceae	palo caja, palo monte	twá padol	le	tr in water, bath	to clean and refresh the skin	●
				st	dec or soaked in rum, ing	to take away the evil from the person	●
Aloe vera L. (8270)	Aloaceae	sabila	lalwá	le (inner part)	ing	internal infections, hepatitis	●
					vaginal suppository	vaginal infections	●
Aloysia triphylla (L'Her) Britton (8267)	Verbenaceae	yerba luisa		le	dec, ing	diarrhea	●
Alpinia speciosa Schum (8600)	Zingiberaceae	colonia	fey canel	le	dec, bath	to clean the skin, ritual bath	●
				ro	dec, ing	catarrh, fever	●
Amaranthus spp. (5198)	Amaranthaceae	bledo	zepiná	le	boiled and eaten as vegetables	intestinal parasites	●●
Ambrosia peruviana Willd. (8468)	Asteraceae	artemisa		le	dec, ing	intestinal parasites	●
Anacardium occidentale L.	Anacardiaceae	marañon	pòm	le	dec, ing	cough, *empacho*	●
				ba	dec, ing	menstrual disorders	●

Annona cherimolia Mill. (7702)	Annonaceae	chirimoya		le	dec, ing	*empacho*	●
Annona muricata L. (8259)	Annonaceae	guanabana	kogosol	le	inf, ing	stenghten the children, for stomach problems of children, catarrh, sedative	●
					dec with leaves that already fell from the tree, ing	high pressure	●
Annona squamosa L. (8261)	Annonaceae	anón		le	dec, ing	*empacho*	●
					mix 16	mix 16	
				ro	mix 17	mix 17	
Artemisia absinthium L. (8617)	Asteraceae	incienso ajenjo	tifey, lapsént	ap	dec or mac in rum, ing	aphrodisiac, it gives strenght to men, intestinal parasites, stomachic	●●
					dec, bath	to clean people and houses for the end of year	●
Bidens pilosa L. (8404)	Asteraceae	romerillo		le	inf, gargles	throat ache	●●
					inf, ing	fever, catarrh, stomach ulcera	●●
					mix 1	mix 1	
					mix 4	mix 4	
Bixa orellana L. (8279)	Bixaceae	bija	woukou	se	smashed and heated with oil, applied as a plasture	burns	●
Boldoa purpuracens Cav. (8395)	Bignoniaceae	nitro		le	dec, ing	diuretic	●
Brachiaria purpurascens (Raddi) Henrard (8280)	Poaceae	paraná		ro	dec, ing	intestinal parasites	●
					mix 19	mix 19	
Bromelia pinguin L.	Bromeliaceae	piña raton	pengwé	fr, inner part	ing at fast in the morning after leaving the fruit some days outside without the peel	intestinal parasites	●

Bursera simaruba L. (8295)	Burseraceae	almacigo	gomyé	le	picked up and put under the pillow	colds, influenza	●
					dec, bath	measle	●●
				sh	mix 17	mix 17	
Caesalpinia bahamensis Lam. (8292)	Fabaceae	brasilete		ba	mac in rum, ing	diuretic	●
Cajanus cajan (L.) Millsp. (8615)	Fabaceae	frijol gandul, gandul	pwa congó	ap	dec, two or three times per day	intestinal parasites	●
					mix 19	mix 19	
				sh	three shoots, dec with salt	*empacho*	●
Canavalia ensiformis (L.) DC. (7864)	Fabaceae	mate	pwa maldjók	se	as collar for children	It protects against the evil eye and helps dentition	●
Canella winterana (L.) Gaertn	Canellaceae	canela	canel	ba	dec or mac in rum, ing	stomach pains, colds	●●
					mix 14	mix 14	
Capraria biflora L. (8458)	Scrophulariaceae	magüiro		sh	dec, ing	colds, inflammations, ovaries pains	●
					mix 20	mix 20	
Carica papaya L. (2704)	Caricaceae	papaya, fruta bomba	papay	young fr	peeled and grated, juice is filtered and drunk for three days with salt	intestinal parasites	●●
				ro	mac in water, drunk as common water	high pressure	●
				se	eaten along with the fruit	to clean kidneys and ovaries	●
Cassia fistula L.	Fabaceae	caña fistula	(kas dou)	se	tr, dec with sugar, ing	anemia	●
Cecropia schrebiana Miq. (8297)	Cecropiaceae	yagruma	tronpèt	yl	dec, ing	asthma, catarrh, cough	●●
					mix 12	mix 12	
Chamissoa altissima (Jacq.) Kunth (8123)	Amaranthaceae		lyann panye	le	mac in rum or dec, ing	aperitive	●

Chenopodium ambrosioides L. (8256)	Chenopodiaceae	apasote	simen kontrá	le	jex, ing with milk in the morning; dec, ing; tr with coconut milk, ing	intestinal parasites	●●●
				sh	roasted, powdered, top	eczema	●
Chiococca alba L. (8236)	Rubiaceae	bejuco verraco, palo verraco	(kok souri)	le	dec in water or mac in rum	aphrodisiac	●
				ro	dec in water or mac in rum	aphrodisiac	●
Chrysophyllum oliviforme L. ssp. *oliviforme* (8143)	Sapotaceae	caimitillo	ti kaymit	le	mix 7	mix 7	
Cissampelos pareira L. (8248)	Menispermaceae		pat chwal	le	dec of three halves of three leaves, ing	fever, stomach pains	●
Cissus verticillata L. (8244)	Vitaceae	bejuco ubí	lyann motel, lyann mol	le	dec with sugar, ing as syrup	catarrh, respiratory problems	●●●
				st, ap	burnt, smoke inhalations	asthma	●
				ap	mix 1, 2, 3, 5	mix 1, 2, 3, 5	
Citrus aurantiifolia (Christm.) Swingle (8284)	Rutaceae	limon	sitron	le	dec, bath	bath for children	●
				fr ju	mix 1	mix 1	
				sh	mix 8	mix 8	
Citrus aurantium L. (8272)	Rutaceae	naranja agria	zoranj si, zoghet	fr	jex with salt and sugar, ing	to clean ovaries	●
				fr peel	mix 10	mix 10	
Citrus limetta Risso	Rutaceae	lima	vergamot	fr peel	inf, ing	high pressure	●
Citrus sinensis (L.) Osbeck	Rutaceae	naranja	zogai, zoranj	fr peel	dec, ing	high pressure, colds	●●
				le	dec, ing	high pressure, colds	●
Cocos nucifera L.	Arecaceae	coco	kok	young fruit	opened, part inside is grated, salt added, ing	to clean ovaries	●
				fr	inner mass is grated and filtered, ing of the resulting liquid for three days at fast in the morning	intestinal parasites	●●●

				ro	mac in rum	diuretic	●
Commelina elegans H.B.K. (8398)	Commelinaceae	canuto		le	mix 7	mix 7	
Corchorus siliquosus L. (8399)	Tiliaceae	malva dulce	valedú	le	dec, filtered, washes	vaginal washes after delivery	●
Coriandrum sativum L.	Apiaceae	cilantro	koulant	le	dec or inf, ing	fever, vomiting	●
Costus speciosus Smuts (8257)	Costaceae	caña mexicana		le	dec, ing	fever	●●
Crescentia cujete L. (8287)	Bignoniaceae	güira	kalbaz	fr	cooked with sugar, honey, and rum (*miel de güira*), ing, mix 12, 13, 14, 15, 16	catarrh, 'cold uterus', stomach problems, intestinal parasites, mix 12,13,14,15,16	●●●
Cucurbita maxima Duch. (9820)	Cucurbitaceae	calabaza	romú	le	dec, ing	*empacho*	●
					mix 6	mix 6	
Cuminum cyminum L.	Apiaceae	comino		se	mix 9	mix 9	
Cymbopogon citratus (DC.) Stapf. (8290)	Poaceae	calentura, cañasanta, yerba de limon	sitwonel	le	dec or inf, ing	*frialdad* (colds), catarrh, stomach problems	●●●
					mix 10, 14	mix 10, 14	
Desmodium incanum (Sw.) DC. (8431)	Fabaceae	amor seco, panadillo	wsé tuasú	ap	dec, ing	ovaries pains	●
					mix 11	mix 11	
Dichrostachys cinerea (L.) Wight & Arn. (5816)	Fabaceae	marabú		le	three leaves, dec, ing	diarrhea	●
Erythroxylum havanense Jacq. (8255)	Erythroxylaceae	jibá		le	dec, ing	catarrh, infections to kidneys	●
				ro	mac in rum or dec, ing	diuretic, respiratory and venereal infections, oedemas	●●
					mix 11	mix 11	
Eucalytus sp.	Myrtaceae	eucalipto		le	dec, ing	catarrh	●
Eupatorium odoratum L. (8620)	Asteraceae	rompezaragüey	lang chat	sh	dec of three shoots with salt, ing; mix 1	catarrh, cough, fever; mix 1	●

				le	dec, bath	bath for children (for end of the year, with a stem of *Artemisia absinthium*) and for measle; to clean from bad spirits the house	●●
					crushed and mixed with honey, ing	asthma	●
Gliricidia sepium (Jacq.) Kunth ex Walp. (8403)	Fabaceae	piñon	meznié	le	dec, ing	catarrh	●
				sh	mix 8	mix 8	
Gossypium barbadense L. (8425)	Malvaceae	algodón	koton	sh	dec, ing	galactogen, catarrh, cough	●
				st	heated at one extreme, collect with a feather the foam at the other extreme and apply topically	to eliminate scars left from burns	●
Gouania polygama (Jacq.) Urb. (8197)	Rhamnaceae	bejuco de indio	lyann savón	st	tr, water is poured on it, and foam is applied topically	burns	●
Guarea guidonia (L.) Sleumer (8278)	Meliaceae	yamagua	majó	fl	dec, ing	catarrh, colds	●
Guazuma ulmifolia Lam. (6277)	Sterculiaceae	guasima, guasima macho	bwa dóm	sh, se	three shoots and five seeds, or three seeds, dec with salt, ing	*empacho*	●
				le	dec, ing	catarrh	●●
Hamelia patens Jacq. (8274)	Rubiaceae	ponasí	fey koray	le	dec, left to cool, baths for children	to clean the skin, for skin eruptions and measle	●
Hibiscus elatus Sw. (8277)	Malvaceae	majagua		fl	mix 12	mix 12	
Illicium verum Hook. F.	Illiaceae	aníz estrellado	aní etoilé	fr	mac in rum or dec, ing	stomachic	●
					mix 14	mix 14	
Ipomoea batatas (L.) Lam.	Convolvulaceae	boniato	patát, duz	sh	dec, ing	galactogen	●●

			patát				
Isocarpha atriplicifolia R. Br. (9568)	Asteraceae	manzanilla	manzaní	ap	dec is filtered and applied topically in drops	eyes infections, conjuntivitis	●
					dec, bath	skin eruptions	●
					dec or inf with salt, ing; mix 18	*empacho*, stomach problems; mix 18	●●
Jatropha gossypifolia L.	Euphorbiaceae	tuatua, tubatuba	timapú	le	dec, ing as common water	colds, inflammations	●
				ro	mix 11, 21	mix 11, 21	
Jatropha urens L. (8265)	Euphorbiaceae	kiaya, chaya		sh	dec, three shoots for three times per day, ing	intestinal parasites	●
Justicia pectoralis Jacq. (8607)	Acanthaceae	tilo	sèpantye	ap	mix 22	mix 22	
Kalanchoe pinnata (Lam.) Pers.	Crassulaceae	hoja de viento, prodigiosa	(lou garou)	le	dec, ing	fever	●
Lawsonia alba L. (9567)	Lythraceae	resedá	(resedá)	le	mix 22	mix 22	
Lepidium virginicum L. (7699)	Brassicaceae	mastuerzo	kreson	le	dec, ing	intestinal parasites, carminative, diuretic, menstruation pains	●●
					heated and applied top on the stomach	to expell gases, carminative	●
Lippia alba (Mill.) N.E. Br. (8239)	Verbenaceae	quitadolor, menta americana	fey melis	ap	dec, ing	catarrh, colds, stomach pains, menstrual pains	●●●
Luffa aegyptiaca Mill. (8633)	Cucurbitaceae	estropago	fey kosción	fr	boiled, washes with the dec	scabies	●
Majorana hortensis Moench (8433)	Lamiaceae	mejorana		ap	inf, ing	stomach pains	●
				le	mix 7	mix 7	
Mangifera indica L.	Anacardiaceae	mango	mangó	le already fallen from the tree	dec or inf, ing	fever, catarrh, stomach problems	●
				yl	dec of three yl, in three different cups, ing	*empacho*	●

				ba	cut in pieces, dec, ing	gastritis, ulcera, refreshing and depurative	●●
Maranta arundinacea L. (8222)	Marantaceae	yuquilla		rh	tr, top appl	fractures, bruises	●
Melia azederach L.	Meliaceae	paraís	(paraís)	le	dec or mac in water, bath	to clean (both ritually and skin cleaning), and take away bad influence	●
Melicoccus bijugatus Jacq. (8258)	Sapindaceae	anoncillo	kenép	le	dec, ing	catarrh	●
Mentha piperita L. var. *citrata* (Ehrh.) Brig. (8613)	Lamiaceae	yerba buena		le	inf in water or mac in rum, ing	stomachic, catarrh	●
Mirabilis jalapa L. (8266)	Bignoniaceae	maravilla		fl	rubbed top	pains	●
Momordica charantia L. (8432)	Cucurbitaceae	cundeamor	asorosí, yeské	le	jex or dec, ing fasting in the morning (sometimes with sugar)	intestinal parasites, blood cleansing, fever, stomach problems	●●●
					dec, or leaves put directly in the water of the bath, baths for children	refreshing, to clean the skin	●●
					dec, add water, ing	drink for diabetic people	●
					juice applied topically to the skin	carbuncles, eruptions of measle and herpesvirus	●
Morinda royoc L. (8235)	Rubiaceae	palo garañon		ro	mac in rum, ing	diuretic, aphrodisiac	●
Mucuna pruriens (Stickm.) DC. ssp. *deeringiana* (Bort) Hanelt (8471)	Fabaceae	nescafé	(pwa grate)	se	dec, also as coffee substitute, ing	stomach problems	●
				hair of the fruit	mixed with guava jam, ing	intestinal parasites	●
Mucuna urens (L.) DC. (8343)	Fabaceae	ojo de buey		se	kept in the pocket	against the evil eye	●
Musa x paradisiaca L.	Musaceae	platano macho	banán	le	dried, dec, ing	*empacho*	●
Myristica fragrans Houtt.	Myristicaceae	nuez moscada	mizká	se	dec or mac in rum, ing	stomach pains, carminative, colds	●●
					mix 14	mix 14	

Ocimum basilicum L. (8443)	Lamiaceae	albahaca blanca	fey bazilik	le	inf, ing	colds, influenza	●●
Ocimum gratissimum L. (8445)	Lamiaceae	albahaca cimarrona o mondonguera	(fonbazen)	le	inf, ing	catarrh	●
Opuntia ficus-indica (L.) Mill.	Cactaceae	tuna	rakèt	le	a piece of the size of a foot is peeled and hanged above the fire, the liquid produced is thrown away, and leaf is then opened and applied topically in front and behind the liver	liver problems	●
Oxalis violacea L. (8743)	Oxalidaceae	trebol		le	dec, mixed with animal fat, ing	asthma	●
Parthenium hysterophorus L. (8255)	Asteraceae	escoba amarga	(lapsént)	ro	mix 20	mix 20	
Pedilanthus tithymaloides (L.) Poit. (8251)	Euphorbiaceae	itamo real		le	jex, top application in drops	skin ulcera, earache	●
Persea americana Mill. (8457)	Lauraceae	aguacate	zaboká	le, collected dry from the soil	dec with salt, ing; mix 6	*empacho*; mix 6	●●
				le	dec, drank as water	arthritis, rheumatic pains	●
				se	grated, dec, applied as shampoo	lice	●
Petiveria alliacea L. (8248)	Phytolaccaceae	anamú	malpughí, mal pourri	le	dec, ing	colds, catarrh, kidney infection	●
					mix 5	mix 5	
Phoradendron quadrangulare (H.B.K) Kr. et Urb. (8355)	Loranthaceae	palo caballero	uàùà	ro	mac in rum, ing	aphrodisiac	●
Phyla scaberrima (Juss. ex Pers.) Moldenke (8474)	Verbenaceae	oro azul		le	dec, ing	fever, ovaries pains	●

Phyllanthus procerus Wr. ex Sauv. (8288)	Euphorbiaceae		deedó	ap	dec, three times per day before fever raises, ing	fever, malaria	●●
					mix 2	mix 2	
Pimenta dioica (L.) Merr. (8289)	Myrtaceae	pimienta dulce	pwa dus, pimá duz	le	inf, ing	stomach problems, colds	●●●
					mix 14	mix 14	
Piper aduncum L. (8247)	Piperaceae	platanillo	fey lesan	le	dec, ing	catarrh	●
					heated and applied top on the forehead for one hour	headache	●
					mix 3	mix 3	
Piper auritum Kunth (8436)	Piperaceae	anisón		le	heatede on the fire and then applied top on the stomach	to expell intestinal gases	●
Plantago major L. (8478)	Plantaginaceae	llantén	plantén	le	dec, ing	fever, kidney problems, ovaries pains	●
					jex, top application in drops	earache, ear infections	●
Pluchea carolinensis (Jacq.) G. Don (8282)	Asteraceae	salvia de playa	tabak mau	le	dec, ing	catarrh, headache	●
					dried and rolled, smoked	respiratory problems (*frialdad*), headache	●
					dec, bath	measle	●
					mix 1	mix 1	
Portulaca oleracea L.	Portulacaceae	verdolaga	kupyé	le	dec or inf, ing	*empacho*	●
					boiled and eaten	intestinal parasites	●●●
Priva lappulacea (L.) Pers. (8599)	Verbenaceae		usevá, kusevá	sh	dec, ing	stomachic, carminative	●●
Protium cubense (Rose) Urb. (8130)	Burseraceae	copal	kopál	le	inhalations, vapours (in the night, without going out afterwards)	catarrh, fever	●
					mix 4	mix 4	

Pseudelephantopus spicatus (Juss.) Rohr. (8601)	Asteraceae	lengua de vaca	lambef (lang vach)	le	rubbed on the skin, top	itching, insect bites	●
					dec with salt, ing	*empacho*, fever	●
Psidium guajava L.	Myrtaceae	guayaba	guayav	sh	dec, three shoots with salt, ing	diarrhea, stomach problems, stomach pains	●●
				le	dec, left to cool, baths for children	to clean the skin and treat skin infections	●●
				young fruit	ing	intestinal parasites	●●●
				sh	mix 8	mix 8	
Rheedia aristata Griseb. (8273)	Clusiaceae	manajú		le	dec, ing	asthma, catarrh	●
				re	top appl	to take out spines	●
				ba	mix 9	mix 9	
Ricinus communis L.	Euphorbiaceae	higuereta	masketí	se	oil, ing	laxative, clean the interior, *empacho*	●
				le	dec, vaginal steam bath by sitting above the pot with the decoction	problems of uterus, ovaries pains	●
					one leaf is tied to the forehead for one night	headache ('the leaf absorbs the headache while getting dry')	●
Senna alata (L.) Roxb. (8275)	Fabaceae	palo santo		le	dec or jex, bath	to clean (both ritually and skin cleaning), and to take away bad influence	●
Senna obtusifolia (L.) Roxb.	Fabaceae	guanina, platanillo	pwa pian	se	dec, toasted and milled, as substitute of coffee, ing	good for blood, headache, stomach pains, anemia, in the morning fasting for diabetes	●●
				ro	grated in a spoon of breast milk, ing	colics of infants	●

Solanum americanum Mill. (5174)	Solanaceae	yerba mora	lamá	le	triturated, juice applied topically	skin ulceras	●
					chewed	aperitive, stomach ulcera	●●
					juice mixed with honey, mouth washes	mouth infections and inflammations	●
Solanum erianthum D. Don. (8628)	Solanaceae	pendejera	zamoghét	le	tr, jex, top application in the eyes	blurred vision	●
					jex, ing	stomach problems, good for kidneys	●
Solanum melongena L.	Solanaceae	berenjena	berenjen	sh, fl	three shoots and three flowers, dec, ing	stomach pains	●
Stachytarpheta jamaicensis (L.) Vahl (8249)	Verbenaceae	verbena	vevén	le	three leaves, dec, drunk as decoction or as common water at fast in the morning	intestinal parasites, to clean the blood	●●
					dec, or leaves put directly in the water of the bath, baths for children and infants	refreshing, to clean the skin, for carbuncles and eczemas	●●●
					dec, ing	catarrh, to help dentition	●
Tamarindus indica L.	Fabaceae	tamarindo	tomagué	le	dec, ing	high pressure	●
				fr	chewed	high pressure	●
Terminalia catappa L. (7003)	Combretaceae	almendra	zamánd	le	dec, ing; mix 3	fever, catarrh; mix 3	●
Thevetia peruviana (Pers.) K. Schum. (2820)	Apocynaceae	cabalonga	fey sezí	se	kept in the pocket	high pressure	●
Tradescantia discolor L'Hér. (7833)	Commelinaceae	cordoban		le	dec of two leaves with sugar, ing at fast in the morning	tonic	●
Tradescantia spathacea Sw. (8614)	Commelinaceae	barquillo, cordobán	sangloriá	le	heated and applied on the forehead	headache	●

Trichilia glabra L. (8233)	Meliaceae	siguaraya		le	dec, bath	to clean and take away bad influences	●●
Turnera ulmifolia L. (8252)	Turneraceae	marilope		le	dec, ing	diarrhea	●
Urera baccifera (L.) Gaud. (8300)	Urticaceae	chichicate		le	dec, ing	diuretic, good for kidneys	●
Vitex trifolia L. (8308)	Verbenaceae	yo puedo mas que tu	kapapaseó	st, le	dec or jex, bath	to clean and take away bad influences	●
Xanthosoma sagittifolium (L.) Schott	Araceae	malanga	malangá, kalaib	tu	cooked without salt and eaten with milk	stomach ulcera	●●
Zingiber officinale Rosc. (8298)	Zingiberaceae	genjibre	gengián	rh	dec, ing	colds, catarrh, rheumatic pains	●●
					mix 10, 16	mix 10, 16	

[a] Non-recorded names are missing from the table. Names within parenthesis are uncorrect names reported by informants, usually a translation of the Spanish name to Creole.

[b] Part(s) used: ap, aerial part; ba, bark; bu, bulb; ep, fruit epicarp; fl, flowers; fr, fruits; ft, flowering tops; la, latex; le, leaves; ls, leaf stalks; re, resin; rh, rhizome; ro, root; se, seeds; sg, stigma; sh, shoots; st, stems; tu, tuber; uf, unripe fruits; wh, young whorls; wo, wood; wp, whole plant.

[c] Preparation: dec, decoction; fr, frying; inf, infusion; jex, juice extraction; mac, maceration; tr, trituration.

[d] Way of use: ing, ingestion; top, topical application

[e] Quotation frequency: ●: quoted by less than 10% of the informants; ●●: quoted by more than 10% and less than 40% of the informants; ●●●: quoted by more than 40% of the informants.
